# Precision agriculture with YOLO-Leaf: advanced methods for detecting apple leaf diseases

**DOI:** 10.3389/fpls.2024.1452502

**Published:** 2024-10-15

**Authors:** Tong Li, Liyuan Zhang, Jianchu Lin

**Affiliations:** ^1^ College of Agriculture and Forestry Economics and Management, Lanzhou University of Finance and Economics, Lanzhou, China; ^2^ Huaiyin Institute of Technology, Huai’an, China; ^3^ Jiangsu Key Lab of Image and Video Understanding for Social Security, and Key Lab of Intelligent Perception and Systems for High-Dimensional Information of Ministry of Education, Nanjing University of Science and Technology, Nanjing, China

**Keywords:** apple leaf disease, dynamic snake convolution, BiFormer, IF-CIOU, YOLOv8

## Abstract

The detection of apple leaf diseases plays a crucial role in ensuring crop health and yield. However, due to variations in lighting and shadow, as well as the complex relationships between perceptual fields and target scales, current detection methods face significant challenges. To address these issues, we propose a new model called YOLO-Leaf. Specifically, YOLO-Leaf utilizes Dynamic Snake Convolution (DSConv) for robust feature extraction, employs BiFormer to enhance the attention mechanism, and introduces IF-CIoU to improve bounding box regression for increased detection accuracy and generalization ability. Experimental results on the FGVC7 and FGVC8 datasets show that YOLO-Leaf significantly outperforms existing models in terms of detection accuracy, achieving mAP50 scores of 93.88% and 95.69%, respectively. This advancement not only validates the effectiveness of our approach but also highlights its practical application potential in agricultural disease detection.

## 1 Introduction

In the process of apple growth, the impact of diseases on yield and quality is crucial (Yao and Liu, 2024). The presence of diseases not only reduces the yield of apples but also diminishes their quality, leading to economic losses (Li J. et al., 2022; Wang et al., 2022). Therefore, accurate diagnosis of diseases and timely implementation of effective control measures are essential for the development of the apple industry. Leaf diseases are more common on apple trees, and their characteristics are usually more pronounced, making them easier to observe and diagnose. This makes leaf diseases one of the key concerns for fruit growers. By promptly identifying and taking corresponding measures, fruit growers can effectively control the spread of diseases and minimize losses (Bonkra et al., 2021; Chau et al., 2022). The continuous development of target detection technology provides new opportunities for the diagnosis and monitoring of apple diseases. With advanced image processing algorithms and deep learning models, automatic detection and identification of apple diseases can be achieved, thereby improving the accuracy and efficiency of diagnosis. However, in practical applications, there are still challenges in achieving accurate detection of multi-scale diseases by detection networks in unconstrained environments. Therefore, further research and improvement of detection algorithms are needed to enhance their applicability and accuracy, thereby better serving the development of the apple cultivation industry (Ren and Wang, 2024).

In the field of disease diagnosis, traditional methods primarily rely on the expertise of agricultural experts and disease atlases. While these methods may offer effective diagnostic results to some extent, they exhibit strong subjectivity in terms of reliability and timeliness. Such subjectivity may lead to inconsistent diagnostic outcomes, thus impacting the effective control and prevention of diseases (Zhong and Zhao, 2020). To address these issues, researchers have turned to using deep learning visual processing techniques. Among these, SSD (Single Shot Multibox Detector) (Sun et al., 2021) is a commonly used target detection algorithm. This algorithm achieves efficient detection speed and accuracy by performing target detection within a single neural network structure. In apple disease detection, SSD can rapidly and accurately identify disease areas on leaves, providing precise localization information. Another commonly used target detection algorithm is Faster R-CNN (Faster Region Convolutional Neural Network) (Gong and Zhang, 2023). Compared to SSD, Faster R-CNN (Gao et al., 2020)employs a two-stage detection strategy, which better captures target features and achieves more accurate detection results. In apple disease detection, Faster R-CNN can effectively distinguish between different types of diseases and provide more detailed diagnostic results. Additionally, YOLOv5 (Li J. et al., 2022)is an emerging target detection algorithm with simple and efficient characteristics. This algorithm achieves target detection through a single neural network structure, enabling rapid and accurate identification of diseases on apple leaves and providing timely and effective prevention and control advice for farmers. Furthermore, the YOLOv8 algorithm has also demonstrated good performance in apple disease detection (Wang et al., 2022). By introducing deeper network structures and more effective feature extraction methods, these algorithms further enhance their performance in apple disease detection tasks, providing more reliable diagnostic tools for farmers.

Although these target detection algorithms have achieved significant effectiveness in apple disease detection, there are some limitations. Firstly, detection networks are typically trained using sample data collected under constrained conditions, which may lack unconstrained environmental factors such as changes in lighting and shadows, specular reflection of leaves, and interference from similar targets in the background. The lack of consideration for these factors may affect the generalization ability and robustness of detection networks in real-world scenarios. Secondly, due to the complex relationship between the perceptual field and target scale, detection networks designed to detect multi-scale targets may exhibit differences in their ability to detect large and small lesions (Sun et al., 2021; Yu et al., 2022). This difference may lead to instability in performance when identifying lesions of different scales, thus affecting the accuracy and reliability of detection. Additionally, in the YOLO series, traditional Intersection over Union (IoU) methods may not accurately identify the position parameters of lesions. Due to the limitations of IoU methods in terms of bounding box size and position, they may fail to accurately identify the position information of lesions in cases where the lesion boundaries are blurred or partially occluded (Hameed Al-bayati and Üstündağ, 2020; Gargade and Khandekar, 2021). Therefore, further research and improvement of identification algorithms are needed to enhance the accuracy and reliability of lesion position parameters.

Drawing from the identified shortcomings, we introduce YOLO-Leaf, a novel network architecture, as an extensive upgrade to YOLOv8. Specifically, dynamic snake convolution is integrated, enabling adaptive adjustment of convolution kernel shape and size during network training to enhance adaptability across diverse target scales and shapes, thereby bolstering detection performance amidst complex environmental conditions. Additionally, a BiFormer structure is introduced to address multi-scale target detection challenges, featuring two parallel attention modules for processing feature maps of varying scales, facilitating simultaneous attention and fusion of multi-scale feature information, thus enhancing detection accuracy. Furthermore, the IF-CIoU method is proposed for precise localization of diseased leaf positions, leveraging a scale factor, r, to optimize auxiliary bounding box generation, ensuring improved alignment with leaf disease target dimensions, thereby augmenting detection performance and expediting model convergence. This holistic integration of methodological refinements equips YOLO-Leaf with superior performance and utility in apple disease detection.

Our contributions are as follows:

- The contribution of this paper lies in the introduction of a novel network architecture named YOLO-Leaf, which integrates dynamic snake convolution and BiFormer attention structure techniques.
- The IF-CIoU loss is proposed for accurately identifying the position information of diseased leaves. By optimizing the generation of auxiliary bounding boxes, this method enhances detection performance and accelerates model convergence speed, providing more reliable technical support for apple disease detection.
- On the dataset of leaf diseases, our method surpasses current approaches.

The structure of this paper is as follows. In the second section, we introduce related work on plant leaf diseases. In the third section, we provide a detailed explanation of the technical implementation of YOLO-Leaf. In the fourth section, we present comparative analyses, wherein the ablation studies substantiate the efficacy of YOLO-Leaf. Finally, in the conclusion section, we discuss the limitations and future prospects.

## 2 Related work

### 2.1 Machine learning in plant disease diagnosis research

In the field of plant disease diagnosis, the application of machine learning methods is increasingly becoming a key technology for improving diagnostic accuracy and efficiency (Chen et al., 2023; Wang J. et al., 2024). Common machine learning models such as Support Vector Machines (SVM), k-Means Clustering (KMC), Decision Trees (DT), and Random Forests (RF) have been proven effective in simulating human diagnostic processes, and handling and analyzing complex physiological and pathological data of plants (Li et al., 2021; Zhang et al., 2022). For instance, Kapil Prashar and Rajneesh Talwar developed a method for identifying cotton leaf diseases using visual features. They combined MLP with overlapping pooling, as well as k-Nearest Neighbors (kNN) and Support Vector Machines (SVM) for precise classification, achieving an accuracy rate exceeding 96% (Prashar et al., 2019). In another study, Wang and others employed Principal Component Analysis (PCA) to effectively reduce the dimensionality of plant disease data and further enhanced the recognition accuracy for grape and wheat diseases by integrating Back Propagation Neural Networks (BPNN) (Xing et al., 2023). This method enabled them to more effectively identify and classify plant diseases, showcasing the practical value of PCA and BPNN in this domain. Liaghat and his team applied machine learning techniques to detect fatal fungal diseases (Ganoderma) in oil palm plantations, achieving up to 97% accuracy in recognition. This research not only improved the accuracy of disease diagnosis but also demonstrated the potential of machine learning techniques in managing specific types of diseases. Furthermore, studies involving the use of hyperspectral imaging technology combined with SVM also show promise (Omrani et al., 2014). Thomas and colleagues used hyperspectral imaging data combined with SVM for a detailed analysis, effectively detecting late blight in potatoes. This technique not only improved the efficiency of image data usage but also provided a new method for early diagnosis of potato diseases. These studies further confirm that combining traditional machine learning models with advanced imaging technologies can significantly enhance the diagnostic accuracy and operational efficiency for plant disease diagnosis, providing new perspectives and tools for future agricultural disease management.

### 2.2 Deep learning in plant disease diagnosis research

In the field of plant disease diagnosis, the application of deep learning has become a key technology for enhancing detection accuracy and efficiency (Zhang X. et al., 2023; Li et al., 2024). Particularly, Convolutional Neural Networks (CNN), as one of the mainstream models in deep learning, are widely used for processing and analyzing complex plant data. Well-known CNN architectures such as AlexNet, VGG, GoogLeNet, and ResNet have proven their effectiveness in visual recognition tasks (Eunice et al., 2022; Fan et al., 2022). Despite this, the detection of apple leaf diseases in practical applications still faces challenges. Typically, the image datasets used for classification are collected in controlled environments, lacking the complexity of real-world settings, which limits the generalization ability of the models. Additionally, conventional image classification techniques often fail to provide detailed information about the type and precise location of diseases (Zhang et al., 2024). To address these issues, researchers have developed real-time detection models suitable for mobile devices. For example, Mobile End AppleNet-SSD (Gong and Zhang, 2023), based on the SSD framework, can automatically detect multiple types of apple leaf diseases in real-time. Similarly, the lightweight Mobile Ghost Attention-YOLO model has been developed for real-time monitoring (Sun et al., 2021). Furthermore, a novel real-time detection framework combining MASK R-CNN (Li H. et al., 2022) and transfer learning has been introduced to enhance the accuracy and practicality of detection. In broader plant disease classification, an attention mechanism module based on CoAtNet (Gao et al., 2023) has been used, achieving an accuracy rate of up to 95.95% in grape leaf disease classification. Other studies, such as the use of a dual-channel residual network with attention mechanisms, have also demonstrated efficient recognition capabilities in strawberry disease detection (Tian et al., 2021; Pal and Kumar, 2023). In other related research, the introduction of multi-level feature fusion in an improved EfficientNet network, as well as the incorporation of Effective Channel Attention (ECA) and dilated convolution in MobilenetV3, have significantly enhanced the performance and speed of disease recognition (Ajra et al., 2020; Wani et al., 2022).

## 3 Method

### 3.1 Overview of our network

**Figure 1:**
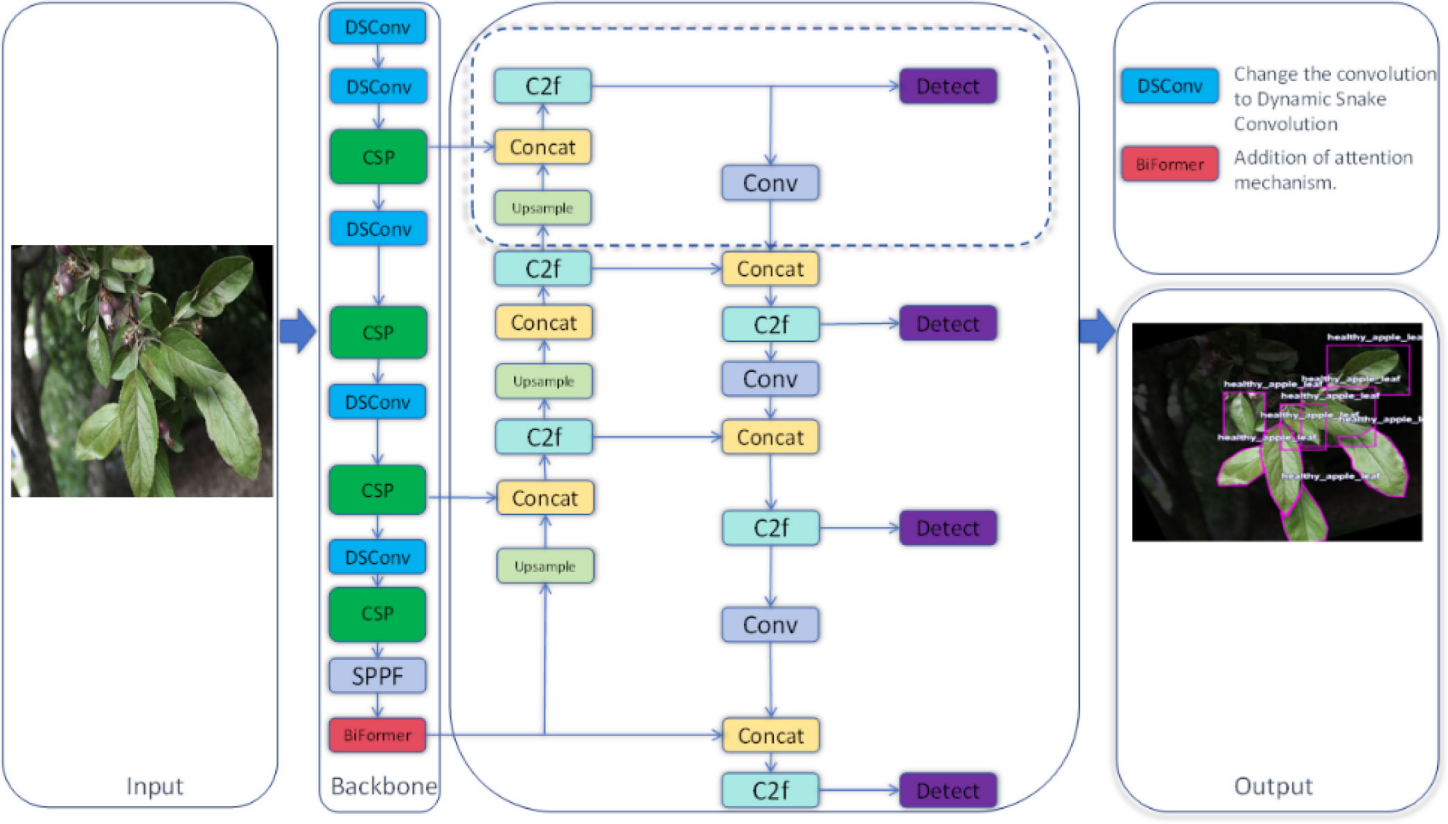
Overall network architecture diagram of YOLO-Leaf.

In this study, we developed a novel deep learning architecture named YOLO-Leaf, which enhances the existing YOLOv8 model for the detection of apple leaf diseases. YOLO-Leaf utilizes a series of innovative technologies to significantly improve the accuracy and efficiency of disease detection in apple leaves. First, we incorporated the Dynamic Snake Convolution developed by Qi et al (Qi et al., 2023), a convolutional mechanism well-suited for handling the twisted and elongated structures found in nature, such as leaves. By adaptively adjusting the shape of the convolutional kernels, it can more precisely capture the detailed changes in critical areas such as leaf veins and edges, thereby enhancing the model’s ability to recognize characteristics of leaf diseases. Secondly, we employed the dual-routing attention structure, Biformer, developed by Zhu et al (Zhu et al., 2023)., which processes feature information from different levels through two parallel attention modules, effectively enhancing the model’s sensitivity and discriminative power at various stages of leaf disease. Additionally, we introduced a new loss function, IF-CIoU, which optimizes the generation of auxiliary bounding boxes through a scaling factor r, taking into account the adaptability of the target frame size to improve the match between the bounding box and the actual leaf targets. These improvements not only enhance the accuracy of object detection but also accelerate the convergence speed of the model, enabling it to respond more quickly and handle the task of detecting leaf diseases. YOLO-Leaf integrates multiple advanced technologies to form an efficient and precise apple leaf disease detection system that provides reliable support in complex agricultural environments, assisting agricultural producers in timely and accurate diagnosis and management of leaf diseases. The overall network diagram proposed in this paper is shown in **Figure 1**.

### 3.2 YOLOv8 network structure

**Figure 2:**
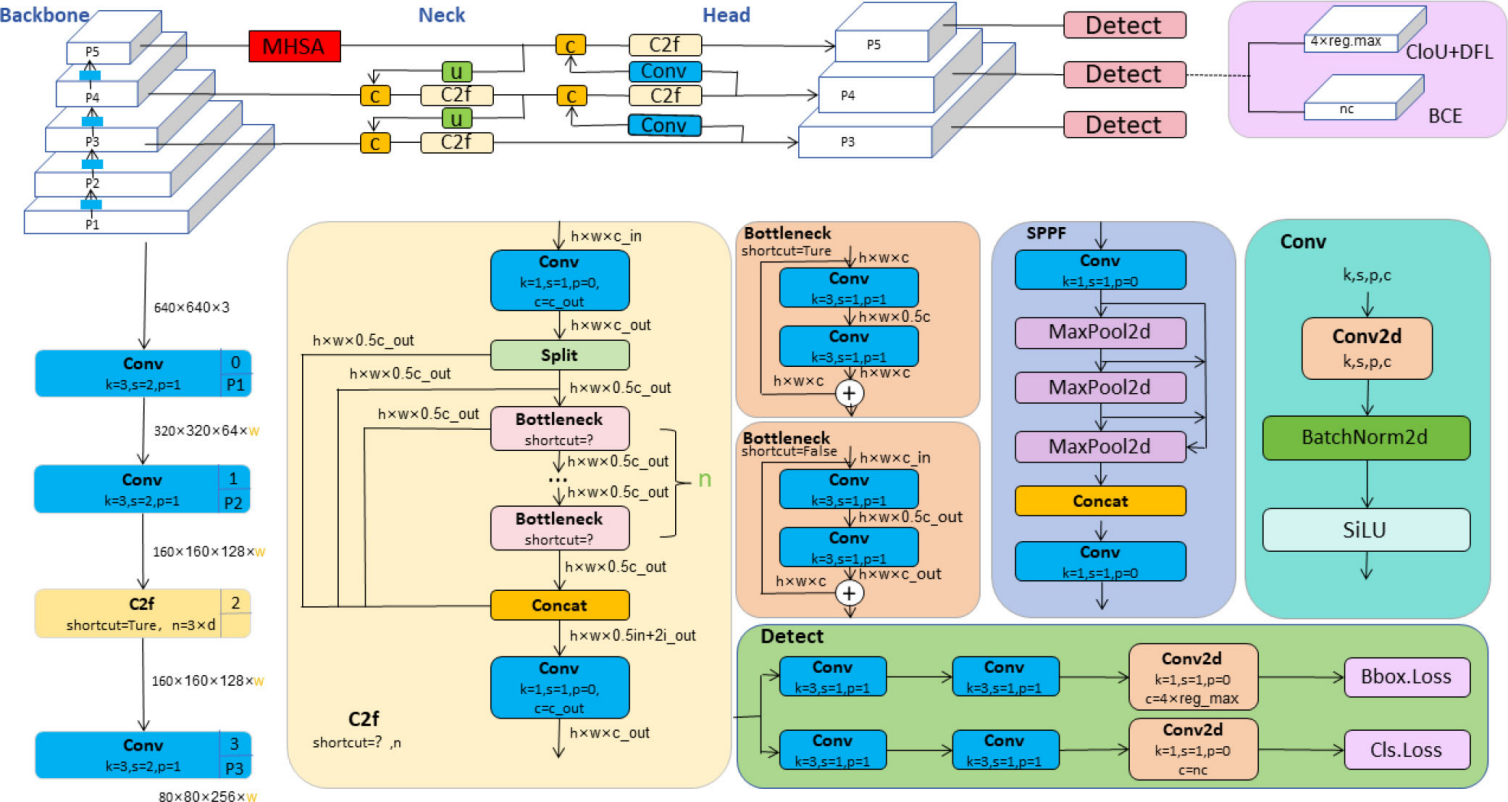
YOLOv8 network architecture diagram (Jocher et al., 2023).

YOLOv8, developed by Ultralytics, represents the latest advancement in the YOLO series of object detection models. (Jocher et al., 2023). This version not only inherits the advantages of its predecessors but also introduces multiple innovations aimed at further enhancing the accuracy, flexibility, and speed of detection. The model is particularly suitable for performing complex real-time visual recognition tasks. In terms of model architecture, the backbone of YOLOv8 adopts the novel C2f structure, an optimization over the C3 structure used in YOLOv5. The C2f structure utilizes a dual-filter cross-convolution method to enhance feature extraction efficiency and precision. At the end of the backbone, YOLOv8 integrates the SPPF (Spatial Pyramid Pooling Fast) module, which extracts multi-level features through various scales of pooling windows, significantly improving the model’s ability to recognize targets of varying sizes. SPPF optimizes the model’s adaptability and stability to changes in target sizes by integrating features from different regions. In the neck part of the model, YOLOv8 uses Concatenation technology to merge feature maps from different scales, a strategy that helps restore spatial resolution that may be lost during downsampling while maintaining important semantic information crucial for precise localization and classification of targets. In the head part, YOLOv8 transitions from a traditional anchor-based design to an anchor-free design, simplifying the model structure, reducing preset parameters, and enhancing the model’s generalization capability and flexibility in new scenarios. The overall network structure of YOLOv8 is illustrated in **Figure 2**.

### 3.3 Dynamic snake convolution

**Figure 3:**
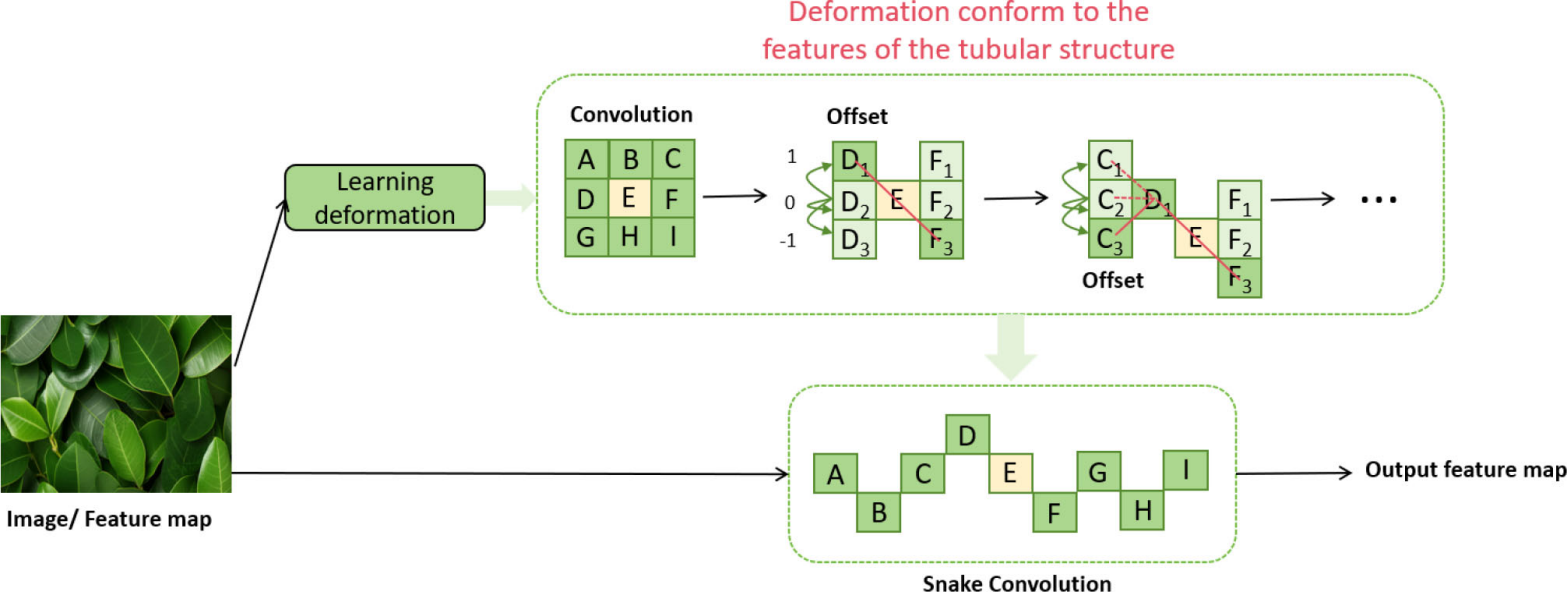
Dynamic snake convolution network architecture diagram (Qi et al., 2023).

In this study, we introduce an innovative convolutional structure named Dynamic Snake Convolution (Qi et al., 2023), proposed by Qi et al., as shown in **Figure 3**. This structure is specifically designed to identify and process complex forms in nature, particularly suited for capturing slender and winding objects such as blood vessels and plant vines. Dynamic Snake Convolution dynamically adjusts the shape and size of the convolution kernels, allowing it to more flexibly adapt to the local features of the target shapes, thereby enhancing the model’s accuracy and efficiency in capturing these complex structures.

In this section, we will discuss in detail how to extract local features of leaf-like structures using Dynamic Snake Convolution. Dynamic Snake Convolution adjusts traditional 2D convolution kernels to better adapt to the curves and twisted forms of the target objects. First, we define a standard 2D convolution kernel *K*, with the center coordinate given by $K_{i}=\;\left(x_{i},y_{i}\right)$ . The formula is summarized as follows:


(1) K={(x−1,y−1),(x−1,y),…,(x+1,y+1)}

In DSConv, the standard convolution kernel is extended along both axes. For a kernel of size 9, we define the positions within the kernel *K* on the x-axis as follows:


(2) $$K_{i\pm c}=\left(x_{i\pm c},y_{i\pm c}\right)$$

where *c* indicates the horizontal distances from the center, ranging from {0,1,2,3,4}. The arrangement of each grid in *K* follows an iterative process, beginning at the central grid $K_{i}$ . The position of each subsequent grid relative to $K_{i}$ is determined by a sequential increment:


(3) Δ={δ|δ∈[−1,1]}

This process necessitates a cumulative adjustment Σ to maintain alignment and consistency across the kernel. The resultant modification on the x-axis is presented as follows:


(4) $$K_{i\pm c}=\{\begin{array}{l} \left(x_{i+c},y_{i+c}\right)=\left(x_{i}+c,y_{i}+\sum_{i=0}^{i+c}\text{Δ}y_{i}\right),\\ \left(x_{i-c},y_{i-c}\right)=\left(x_{i}-c,y_{i}+\sum_{i=0}^{i-c}\text{Δ}y_{i}\right) \end{array}$$

The final change on the y-axis is:


(5) $$K_{j\pm c}=\{\begin{array}{l} \left(x_{i+c},y_{i+c}\right)=\left(x_{j}+\sum_{j=i}^{i+c}\text{Δ}x_{j},y_{j}+c\right),\\ \left(x_{i-c},y_{i-c}\right)=\left(x_{j}+\sum_{j=i-c}^{i}\text{Δ}x_{j},y_{j}-c\right) \end{array}$$

### 3.4 IF-CIoU

To address the issues of poor generalization and slow convergence speed exhibited by existing IOU loss in various detection tasks, this study proposes an innovative method called IF-CIoU (Inner-Focused Complete Intersection over Union). This method improves upon the concept of Inner IOU by using a scaling factor *r* to adjust the size of the auxiliary box. The expression for Inner IOU is as follows:


(6) $$x_{1_{inner}}^{gt}=x_{c}^{gt}-\frac{\left(x_{2}^{gt}-x_{1}^{gt}\right)}{2}*r$$

(7) $$x_{2_{inner}}^{gt}=x_{c}^{gt}+\frac{\left(x_{2}^{gt}-x_{1}^{gt}\right)}{2}*r$$

(8) $$y_{1_{inner}}^{gt}=y_{c}^{gt}+\frac{\left(y_{1}^{gt}-y_{2}^{gt}\right)}{2}*r$$

(9) $$y_{2_{inner}}^{gt}=y_{c}^{gt}-\frac{\left(y_{1}^{gt}-y_{2}^{gt}\right)}{2}*r$$

(10) $$x_{1_{inner}}^{p}=x_{c}^{p}-\frac{\left(x_{2}^{p}-x_{1}^{p}\right)}{2}*r$$

(11) $$x_{2_{inner}}^{p}=x_{c}^{p}+\frac{\left(x_{2}^{p}-x_{1}^{p}\right)}{2}*r$$

(12) $$y_{1_{inner}}^{p}=y_{c}^{p}+\frac{\left(y_{1}^{p}-y_{2}^{p}\right)}{2}*r$$

(13) $$y_{2_{inner}}^{p}=y_{c}^{p}-\frac{\left(y_{1}^{p}-y_{2}^{p}\right)}{2}*r$$

We found that for high IOU samples, the absolute IOU gradient of smaller auxiliary boxes exceeded that of the ground truth IOU gradient (Zhang H. et al., 2023). Based on this observation, using smaller auxiliary boxes for IOU loss calculation can enhance the regression of high IOU samples. Conversely, using larger auxiliary boxes for IOU loss calculation can accelerate the regression process of low IOU samples. The definition is as follows:


(14) $$w_{inner}^{inner}=\text{max }\left(\left(\text{min }(x_{2_{inner}}^{gt},x_{2_{inner}}^{p})-\text{max }(x_{1_{inner}}^{gt},x_{1_{inner}}^{p})\right),0\right)$$

(15) $$h_{inner}^{inner}=\text{max }\left(\left(\text{min }(y_{1_{inner}}^{gt},y_{1_{inner}}^{p})-\text{max }(y_{2_{inner}}^{gt},y_{2_{inner}}^{p})\right),0\right)$$

(16) $$inter_{inner}^{inner}=w_{inner}^{inner}\times h_{inner}^{inner}$$

(17) $$gtunion_{inner}^{inner}=\left(x_{2_{inner}}^{gt}-x_{1_{inner}}^{gt}\right)\times \left(y_{1_{inner}}^{gt}-y_{2_{inner}}^{gt}\right)$$

(18) $$punion_{inner}^{inner}=\left(x_{2_{inner}}^{p}-x_{1_{inner}}^{p}\right)\times \left(y_{1_{inner}}^{p}-y_{2_{inner}}^{p}\right)$$

(19) $$union_{inner}^{inner}=gtunion_{inner}^{inner}+punion_{inner}^{inner}-inter_{inner}^{inner}$$

Given the high proportion of high IoU samples in the leaf disease dataset, this study specifically set r=0.6. With this configuration, the absolute IoU gradient values for smaller auxiliary boxes exceed those at the ground truth boxes. This particular setup helps accelerate the model’s convergence speed, ultimately enhancing its detection performance.

This study introduces IF-CIoU, an innovative loss function for object detection designed to enhance the generalization performance of detection algorithms across various tasks. By incorporating an additional parameter *r*, it allows flexible adjustment within a specified range. The inclusion of a weighted combination in the traditional IOU calculation enables the loss function to be adaptively adjusted. This approach allows the model to better accommodate the scale and shape of objects. The expression of IF-CIoU is as follows:


(20) $$L_{IF-CIoU}=1-{\left(IOU_{inner}\right)}^{r}+{\left(\frac{\rho^{2}}{C^{2}}\right)}^{r}+{\left(r_{2}v\right)}^{r}$$

(21) $$r=\frac{v}{1-IOU+v}$$

(22) $$v=\frac{4}{\pi^{2}}{\left(\text{arctan }\frac{w_{g}}{h_{g}}-\text{arctan }\frac{w_{p}}{h_{p}}\right)}^{2}$$

Where *v* represents the difference in aspect ratio between the predicted and the actual bounding boxes, calculated by scaling the square of the difference between the arctan values of their width-to-height ratios, ensuring that *v* ranges from 0 (indicating no difference) to 1 (indicating the maximum difference). This influences the loss adjustment in the IF-CIoU formula by reflecting shape disparities. *r* serves as a weighting factor within the IF-CIoU loss, adjusting how the aspect ratio differences affect the overall loss calculation, allowing the model to prioritize or reduce the importance of shape alignment depending on the specific detection needs. *ρ* denotes the Euclidean distance between the centers of the predicted and actual bounding boxes, which is crucial for evaluating the positional accuracy of the detection. *C* acts as a normalization factor, often the diagonal length of the bounding box, used to normalize *ρ*, ensuring fair comparisons across different object sizes and avoiding biases towards larger or smaller objects. Together, these variables integrate to finely tune the IF-CIoU loss function for improved object detection performance.

### 3.5 BiFormer structure

**Figure 4:**
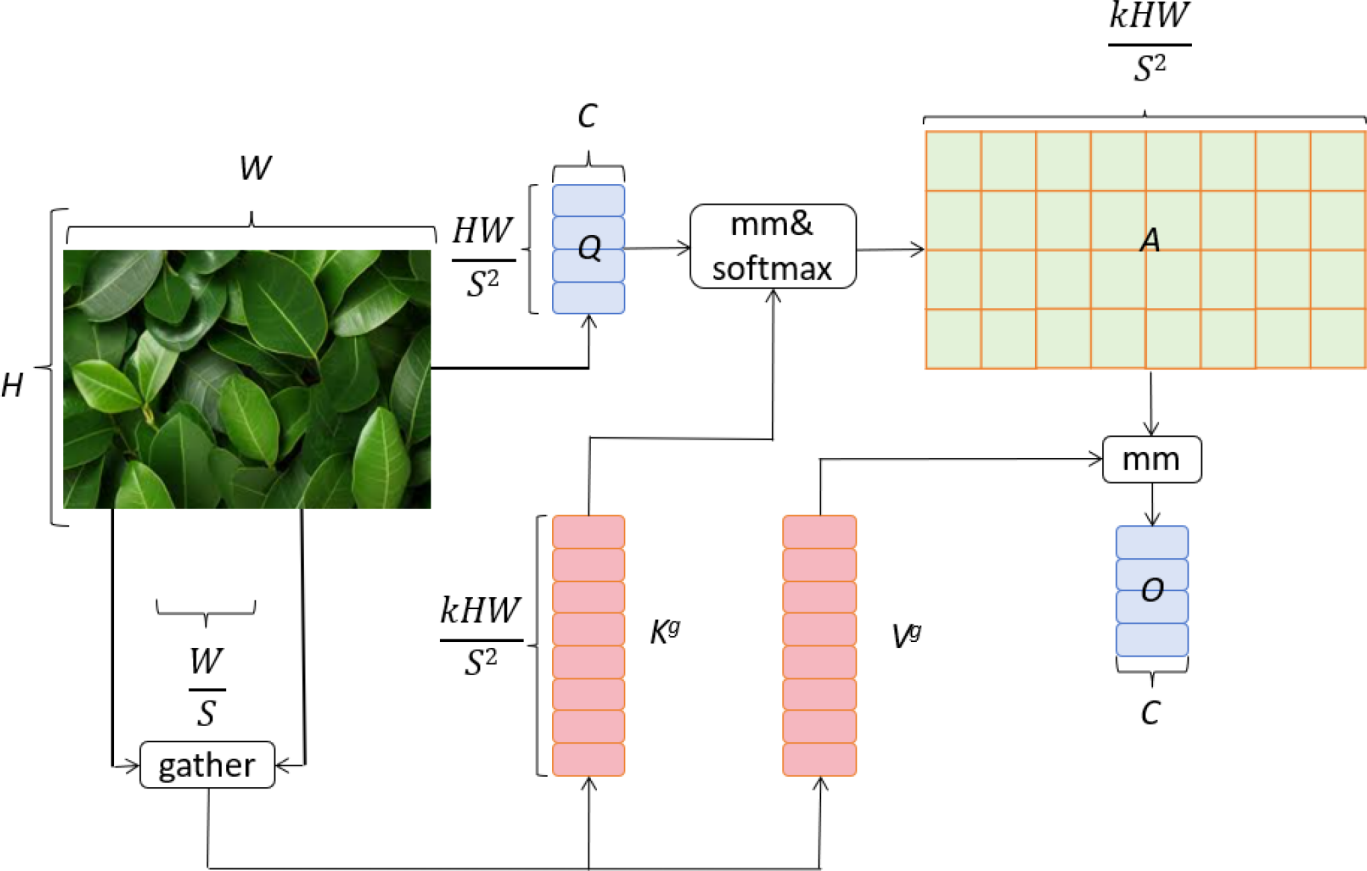
BiFormer network architecture (Zhu et al., 2023).

In this study, we introduce a dual-layer routing attention mechanism based on the vision Transformer model, BiFormer (Zhu et al., 2023). This mechanism enhances feature representation through the interaction between global and local attention layers. The global attention layer captures the overall structure and semantics of the image, while the local attention layer focuses on detailed and localized features. The global attention layer effectively identifies the overall damage and widespread distribution of diseases on apple leaves. This is crucial for assessing leaf health and understanding the extent of disease spread. By weighting each pixel in the image, the global attention layer enables the model to focus on the overall morphology and distribution of the disease. On the other hand, the local attention layer is dedicated to detecting small disease spots and their specific characteristics on the leaves. With higher resolution, the local attention layer captures image details, allowing the model to identify small but critical diseased areas. This detailed capture capability is essential for early detection and diagnosis of diseases. The interaction between global and local attention layers allows the dual-layer routing attention mechanism to effectively integrate global and local information at different scales. This enhances the accuracy and robustness of the apple leaf disease detection model. By combining global and local features, the model can quickly identify obvious disease areas and detect subtle lesions, providing farmers with comprehensive and accurate diagnostic information. The BiFormer network structure is shown in **Figure 4**.

## 4 Experiments

### 4.1 Datasets

In this experiment, we used two public Kaggle competition datasets, namely Plant Pathology 2020-FGVC7 (FGVC7) (Thapa et al., 2020) and Plant Pathology 2021-FGVC8 (FGVC8) (Ait Nasser and Akhloufi, 2024).

**Figure 5:**
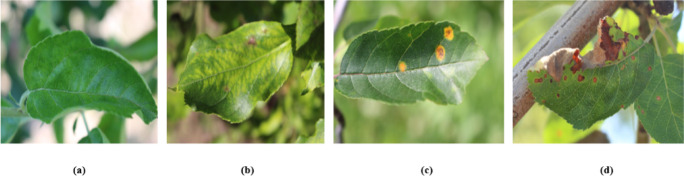
Sample display. **(A)** Healthy. **(B)** Scab.**(C)** Rust. **(D)** multiple diseases.

**Plant Pathology 2020-FGVC7(FGVC7).** This study leverages the FGVC7 dataset (Thapa et al., 2020), consisting of a total of 3642 images. Of these, 2549 images are designated for training, while the test and validation sets each comprise 546 images. The dataset classifies apple leaves into four distinct categories: healthy, apple rust, apple scab, and multiple diseases. **Figure 5** presents exemplars of these classifications, illustrating healthy leaves, leaves afflicted with apple rust, leaves exhibiting apple scab, and leaves manifesting multiple pathogenic conditions.

**Figure 6:**
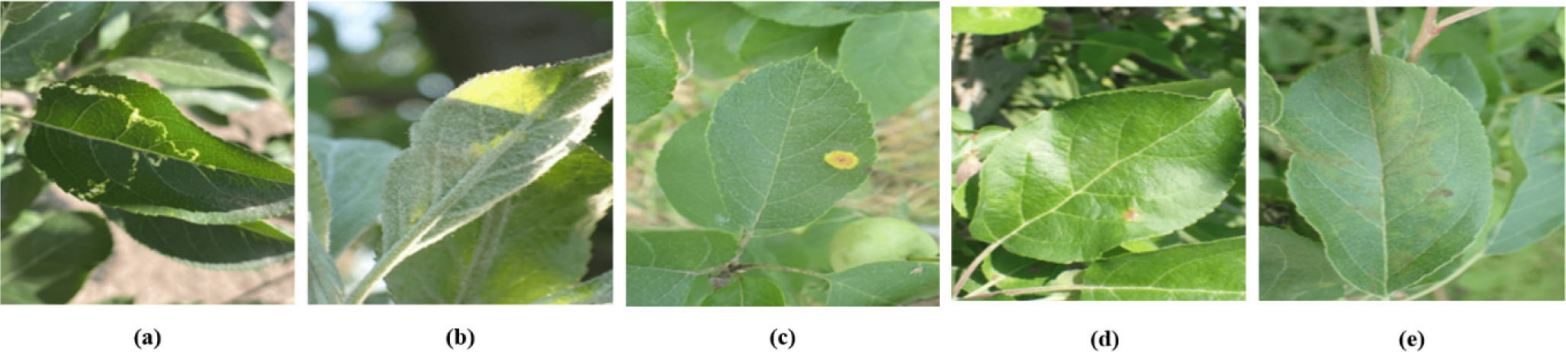
Sample display. **(A)** Mosaic disease. **(B)** Powdery mildew. **(C)** Rust. **(D)** Frog eye leaf spot. **(E)** Scab.

**Plant Pathology 2021-FGVC8(FGVC8).** The FGVC8 dataset (Ait Nasser and Akhloufi, 2024) encompasses a total of 4182 images, stratified into 2927 for training, and 627 each for testing and validation. This dataset characterizes five prevalent apple diseases: black rot, frog eye leaf spot, rust, powdery mildew, and mosaic. **Figure 6** illustrates instances of these five foliar conditions. Given that multiple diseases can manifest on a single leaf, this dataset is particularly well-suited for the simultaneous detection of various pathologies on individual leaves.

We used the LabelImg tool for dataset annotation, saving the results as PASCAL VOC XML files. Agricultural experts manually annotated and verified all images for accuracy. For model training, all images were resized to 640 × 480. **Table 1** shows the specific dataset divisions.

**Table 1:** Dataset division details.

Dataset	Total Images	Training Set	Test Set	Validation Set
FGVC7	3642	2549	546	546
FGVC8	4182	2927	627	627

### 4.2 Implementation details

**Figure 7:**
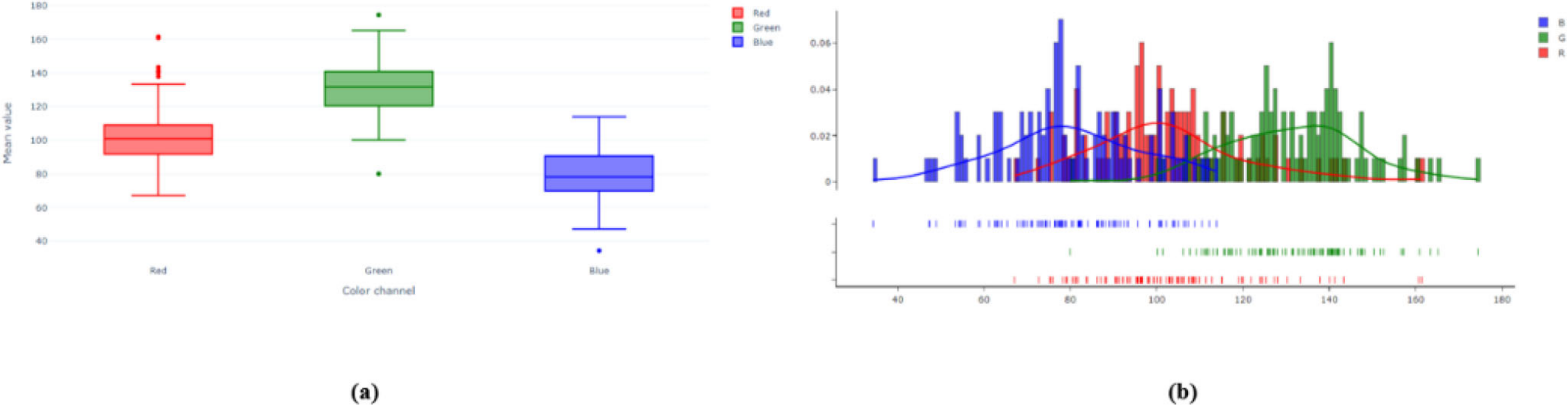
RGB Distribution of the Datasets. **(A)** Plant Pathology 2020-FGVC. **(B)** Plant Pathology 2021-FGVC.

**Data Analysis.** In apple leaf disease detection, color information is crucial for identifying disease types. Different diseases often exhibit distinct color patterns and characteristics on the leaves, making the study and analysis of color space vital for enhancing detection accuracy and robustness. By exploring the RGB distribution in the color space, we can better understand the color characteristics of various diseases, providing valuable insights for model training and optimization. As shown in **Figure 7**, we present the RGB distribution of two datasets. For example, in **Figure 7B**, the red channel exhibits positive skewness, meaning values are more concentrated at intensities below the mean. The green channel shows negative skewness, indicating values are more concentrated at intensities above the mean, making the green channel more prominent in the sample images compared to the red channel. Similarly, the blue channel has slight positive skewness and is well-distributed. These skewness characteristics of the color channels reveal the significance and differences of each channel in the sample images, laying the foundation for further color space analysis.

**Figure 8:**
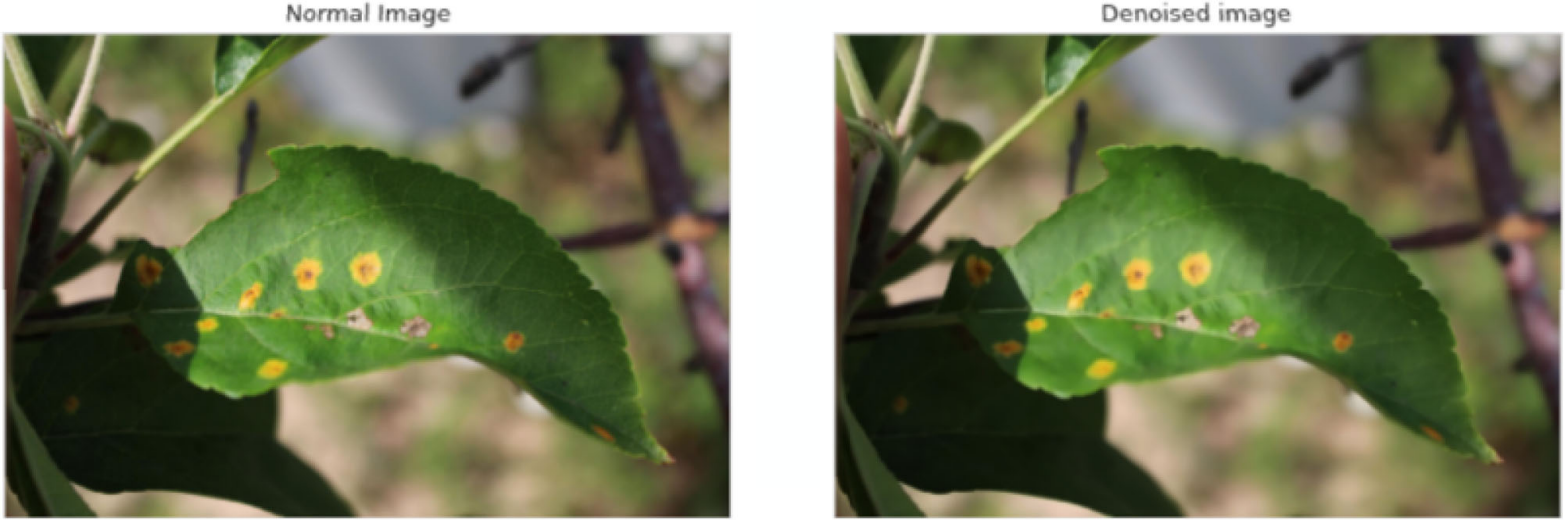
Effect of non-local means denoising on apple leaf images. **(A)** Original noisy image. **(B)** Denoised image.

**Data Preprocessing.** In the context of apple leaf disease detection, our experimental dataset comprises numerous noisy images, which can potentially impair the detection model’s accuracy. To ameliorate noise, we initially employed Gaussian blur, which serves effectively in mitigating minor noise perturbations but shows limitations against more substantial noise. Consequently, a more sophisticated denoising approach was necessitated. In this study, we implemented the Non-Local Means Denoising technique, renowned for its efficacy in noise reduction within images. Specifically, this technique evaluates a small window within the image (e.g., a 5x5 window) and identifies similar patches located elsewhere, potentially within a proximate neighborhood. By averaging these identified similar patches, a superior denoised image can be realized. This approach not only capitalizes on spatial neighborhood similarity but also harnesses resemblance across the entire image, thereby augmenting the denoising effect. Such preprocessing culminates in cleaner images, furnishing higher quality input data for the subsequent apple leaf disease detection phase. **Figure 8** illustrates the denoising process’s effectiveness.

**Model Parameters.** Our parameter settings are summarized as follows: the learning rate is set to 0.001, batch size is 8, weight decay is 0.0002, and the number of epochs is 1000. The network consists of 255 layers and has a total of 11,236,528 parameters, as shown in **Table 2**.

**Table 2:** Training parameters.

Parameter	Value
Learning Rate	0.001
Batch Size	8
Weight Decay	0.0002
Epochs	1000
Layers	255
Parameters	11,236,528

**Experimental Setup.** The experiment was conducted with the following hardware and software configurations: the operating system was Windows 10, the CPU was Intel(R) Core (TM) i9-12950HX, and the GPU was NVIDIA GeForce RTX 3090 with 24G of display memory. CUDA 11.2 and CUDNN V8.0.5 were used for acceleration. The deep learning framework was Pytorch 1.11, the Python version was 3.8, and the development environment was PyCharm 2021. The specific settings are shown in **Table 3**:

**Table 3:** Software and hardware configuration.

Software and Hardware	Version or Model
Operating system	Windows 10
CPU	Intel(R) Core (TM) i9-12950HX
GPU	NVIDIA GeForce RTX 3090
Display memory	24G
CUDA	11.2
CUDNN	V8.0.5
Pytorch version	1.11
Python version	3.8
Software	PyCharm 2021

### 4.3 Metrics

In this experiment, our method evaluates the performance of the model using the following metrics: Precision (PR), Recall (RE), Sensitivity, Specificity, Accuracy, F1-Score, and mAP50.

**Precision (PR)**: Precision is the ratio of correctly predicted positive observations to the total predicted positives.


(23) $$PR=\frac{TP}{TP+FP}$$

**Recall (RE)**: Recall is the ratio of correctly predicted positive observations to all observations in actual class.


(24) $$RE=\frac{TP}{TP+FN}$$

**Sensitivity**: Sensitivity is another term for recall, which is the true positive rate.


(25) $$Sensitivity=\frac{TP}{TP+FN}$$

**Specificity**: Specificity is the ratio of correctly predicted negative observations to all observations in actual negative class.


(26) $$Specificity=\frac{TN}{TN+FP}$$

**Accuracy**: Accuracy is the ratio of correctly predicted observations to the total observations.


(27) $$Accuracy=\frac{TP+TN}{TP+TN+FP+FN}$$

**F1-Score**: F1-Score is the weighted average of Precision and Recall.


(28) $$F1-Score=2\times \frac{PR\times RE}{PR+RE}$$

**mAP50 (mean Average Precision at IoU=0.5)**: mAP50 is the mean of the average precision values for each class, where average precision is computed as the area under the precision-recall curve.


(29) $$mAP50=\frac{1}{N}\sum_{i=1}^{N}AP_{50}^{i}$$

where *TP* is the number of true positives, *TN* is the number of true negatives, *FP* is the number of false positives, *FN* is the number of false negatives, *N* is the number of classes, and $AP_{50}$ is the average precision at IoU=0.5 for class *i*.

### 4.4 Model training

**Figure 9:**
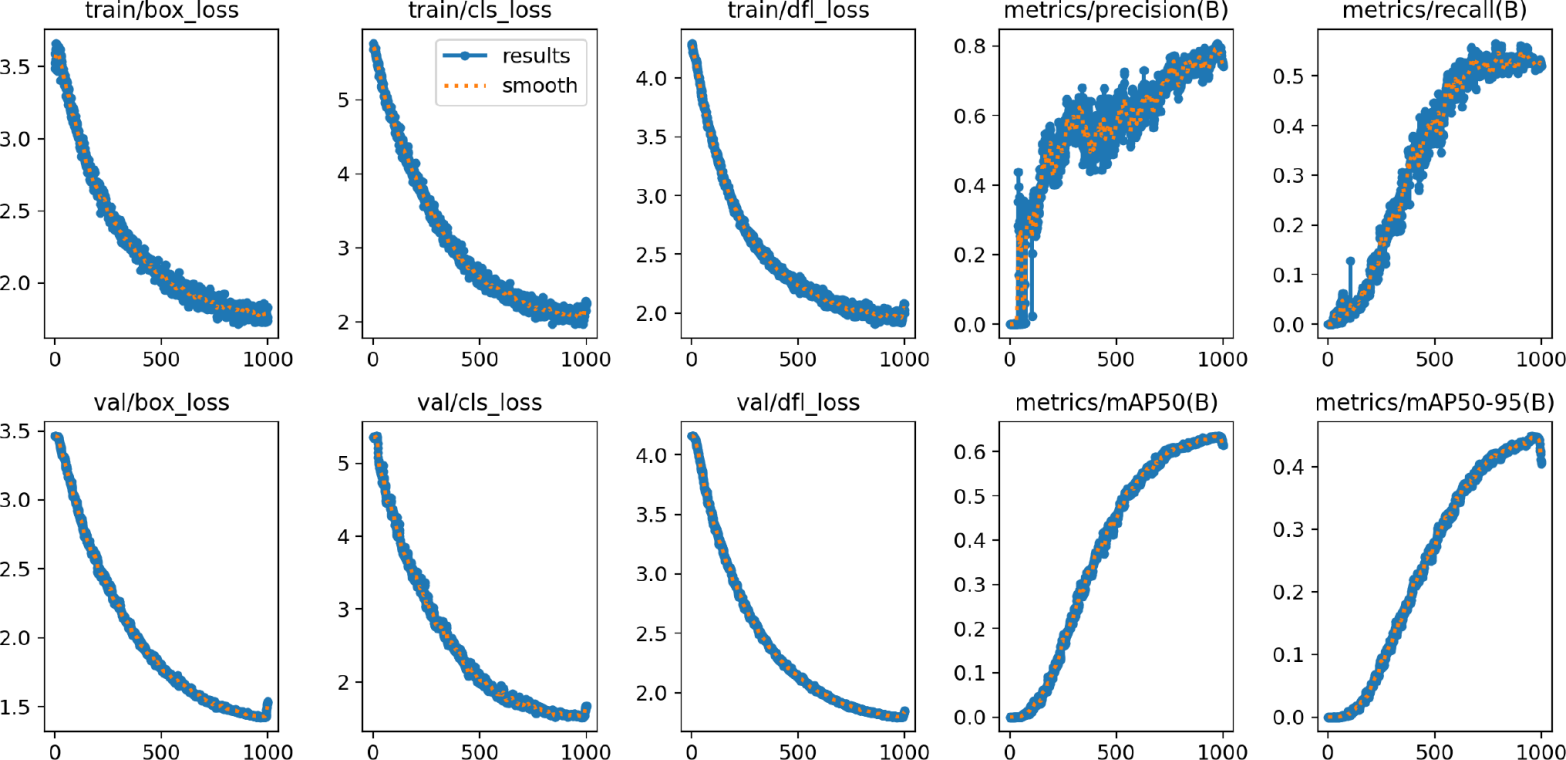
The results of the proposed model.

**Training Results.** **Figure 9** illustrates the changes in the loss function and evaluation metrics during the training and validation phases of the YOLO-Leaf model. The figure shows the bounding box loss, classification loss, and dynamic convolution loss for both the training and validation datasets. From the graph, it can be observed that our model gradually optimizes during training and consistently improves its performance on the validation set. As training progresses, the loss function exhibits a downward trend, indicating that the model is gradually converging. Additionally, the improvement in evaluation metrics demonstrates the model’s effectiveness in feature learning and target recognition.

### 4.5 Result

**Comparison to PriorWork.** As shown in **Table 4**, the performance comparison of different models on the FGVC7 and FGVC8 datasets demonstrates the significant advantages of our method. By analyzing the evaluation metrics in the table, it is clear that our method outperforms other models across all indicators, indicating its superior performance in the task of apple leaf disease detection. On the FGVC7 dataset, our method achieved 93.88% in mAP50, 93.84% in Precision (PR), 93.89% in Accuracy (AC), and 93.80% in F1-Score, significantly higher than YOLOv8. Particularly in the mAP50 metric, our method exceeded YOLOv8 by nearly 1.79 percentage points, demonstrating higher detection accuracy. On the FGVC8 dataset, our method also performed exceptionally well, achieving 95.69% in mAP50, 95.65% in Precision, 95.68% in Accuracy, and 95.62% in F1-Score, again far surpassing YOLOv8. With an increase of 1.80 percentage points in mAP50 over YOLOv8, our method further proves its superiority. It is worth mentioning that YOLOv10 did not achieve high performance in this experiment’s leaf disease detection. These results indicate that our method has higher robustness and generalizability in complex agricultural environments. It not only enhances the accuracy of disease detection but also effectively handles multiple types of diseases, providing more reliable technological support for agricultural producers.

**Table 4:** Comparison of model performance on Roboflow and Br35H datasets.

Models	FGVC7				FGVC8			
	mAP50 (%)	PR (%)	AC (%)	F1-Score (%)	mAP50 (%)	PR (%)	AC (%)	F1-Score (%)
YOLOv3 (Tian et al., 2019)	90.08	89.84	90.04	89.20	91.50	91.64	91.82	92.02
YOLOv4 (Xinming et al., 2023)	90.64	90.54	90.72	90.85	92.42	92.34	92.51	92.59
YOLOv5 (Li J. et al., 2022)	92.02	91.62	90.94	91.11	93.11	92.41	92.71	92.95
YOLOv6 (Krishnarji and Riyazuddin, 2024)	91.14	90.71	90.84	90.34	92.88	92.52	92.65	93.09
YOLOv7 (Ma et al., 2023)	91.84	91.74	92.04	91.94	93.62	93.52	93.80	93.70
YOLOv8 (BalaChandralekha and Thangakumar, 2024)	92.09	91.93	92.24	92.24	93.89	93.73	93.99	93.99
YOLOv10 (Wang A. et al., 2024)	91.05	90.25	90.23	90.14	91.65	90.73	91.55	90.39
Ours	93.88	93.84	93.89	93.80	95.69	95.65	95.68	95.62

**Figure 10:**
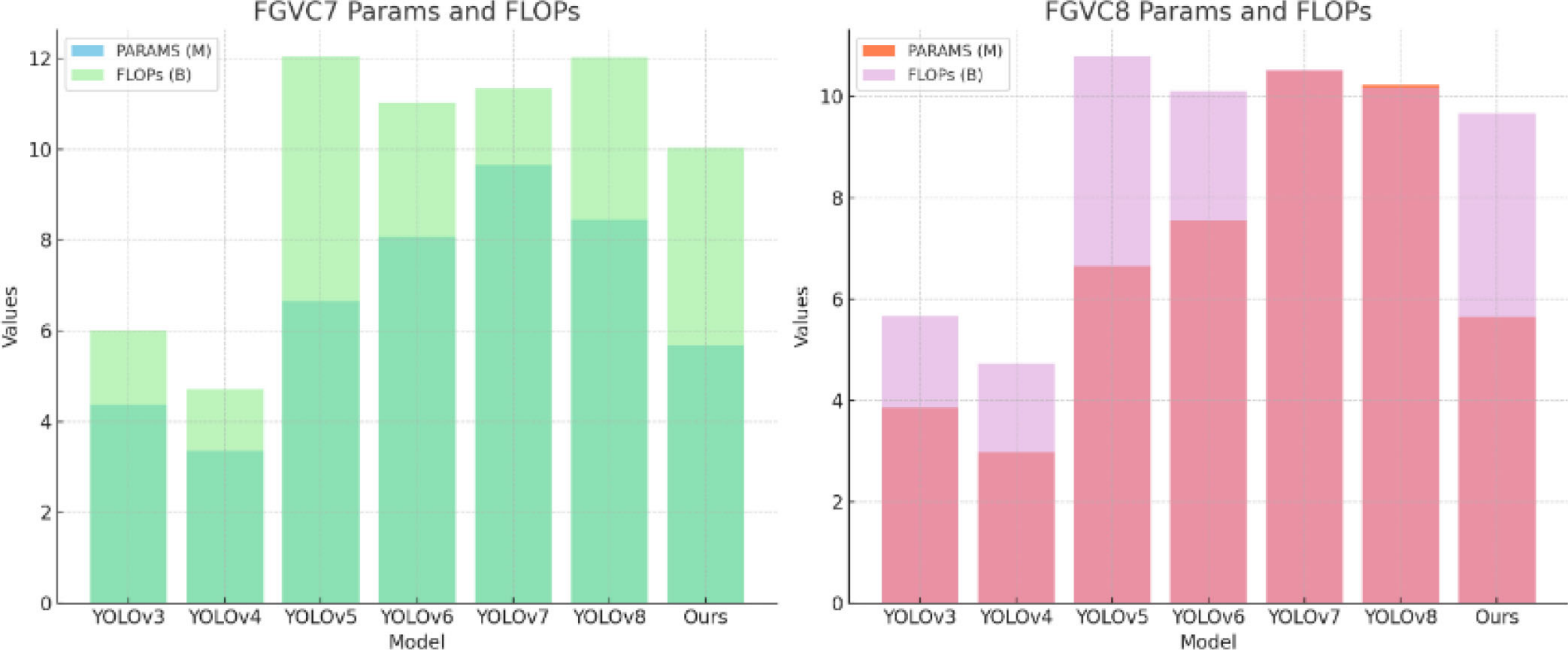
Different model parameter comparisons.

**Parameter Comparisons.** As shown in **Table 5**, the comparison of model parameters (PARAMS) and floating point operations (FLOPs) highlights the performance differences of various models on the FGVC7 and FGVC8 datasets. By analyzing the table, it is evident that our method maintains efficient performance while keeping the parameters and computational load relatively low. In the FGVC7 dataset, our method’s parameters (PARAMS) are 5.68M and the floating point operations (FLOPs) are 10.03B, whereas YOLOv5 has 6.65M parameters and 12.03B FLOPs. Compared to YOLOv5, our method reduces the parameters by 0.97M and the FLOPs by 1.99B, demonstrating an advantage in resource usage. Similarly, in the FGVC8 dataset, our method’s parameters and FLOPs are 5.65M and 9.67B, respectively, while YOLOv8’s parameters and FLOPs are 10.23M and 10.18B. Compared to YOLOv8, our method reduces the parameters by 4.58M and the FLOPs by 0.51B, further proving the efficiency and lightweight nature of our approach. These specific numerical comparisons highlight the advantages of our method, showing that we achieve high performance while significantly reducing the demand for computational resources. **Figure 10** visualizes the content of the table, further illustrating the benefits of our method.

**Table 5:** Comparison of model parameters (PARAMS) and floating point operations (FLOPs) on Roboflow and Br35H datasets.

Model	FGVC7		FGVC8	
	PARAMS	FLOPs	PARAMS	FLOPs
YOLOv3	4.38 M	6.01 B	3.86 M	5.67 B
YOLOv4	3.35 M	4.72 B	2.98 M	4.73 B
YOLOv5	6.65 M	12.03 B	6.65 M	10.78 B
YOLOv6	8.07 M	11.03 B	7.56 M	10.11 B
YOLOv7	9.66 M	11.34 B	10.51 M	10.52 B
YOLOv8	8.45 M	12.02 B	10.23 M	10.18 B
Ours	5.68 M	10.03 B	5.65 M	9.67 B

### 4.6 Qualitative analysis

**Figure 11:**
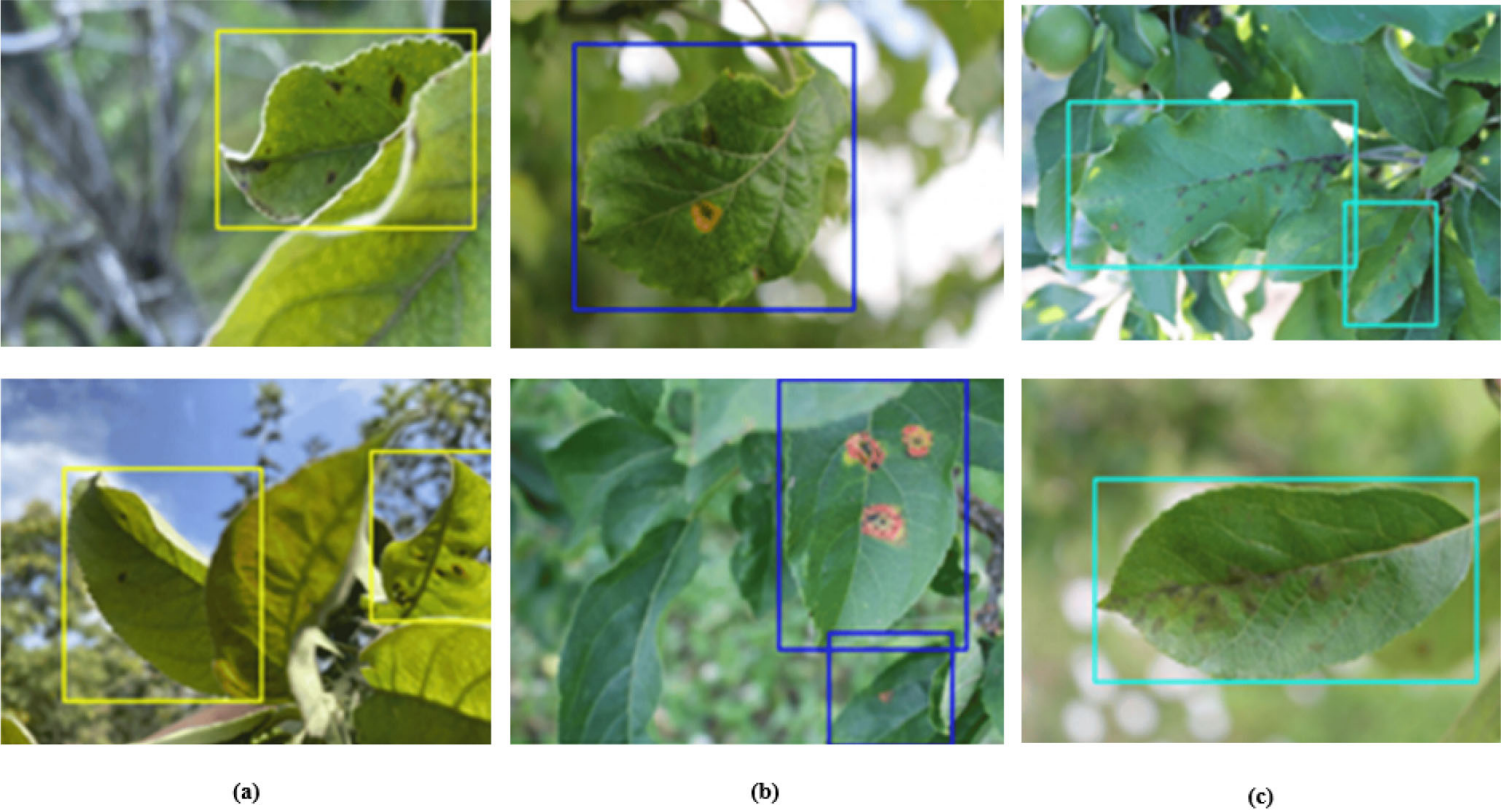
YOLO-Leaf detection results. **(A)** leaf Powdery, **(B)** leaf rust, **(C)** leaf scab.

**Figure 11** visualizes the detection results of our model. From the images, it is evident that our model effectively identifies and classifies different types of leaf diseases, including (a) leaf blotch, (b) leaf rust, and (c) leaf scab. The clear and accurate detections across these various conditions highlight the robustness and precision of our model in detecting apple leaf diseases.

**Figure 12:**
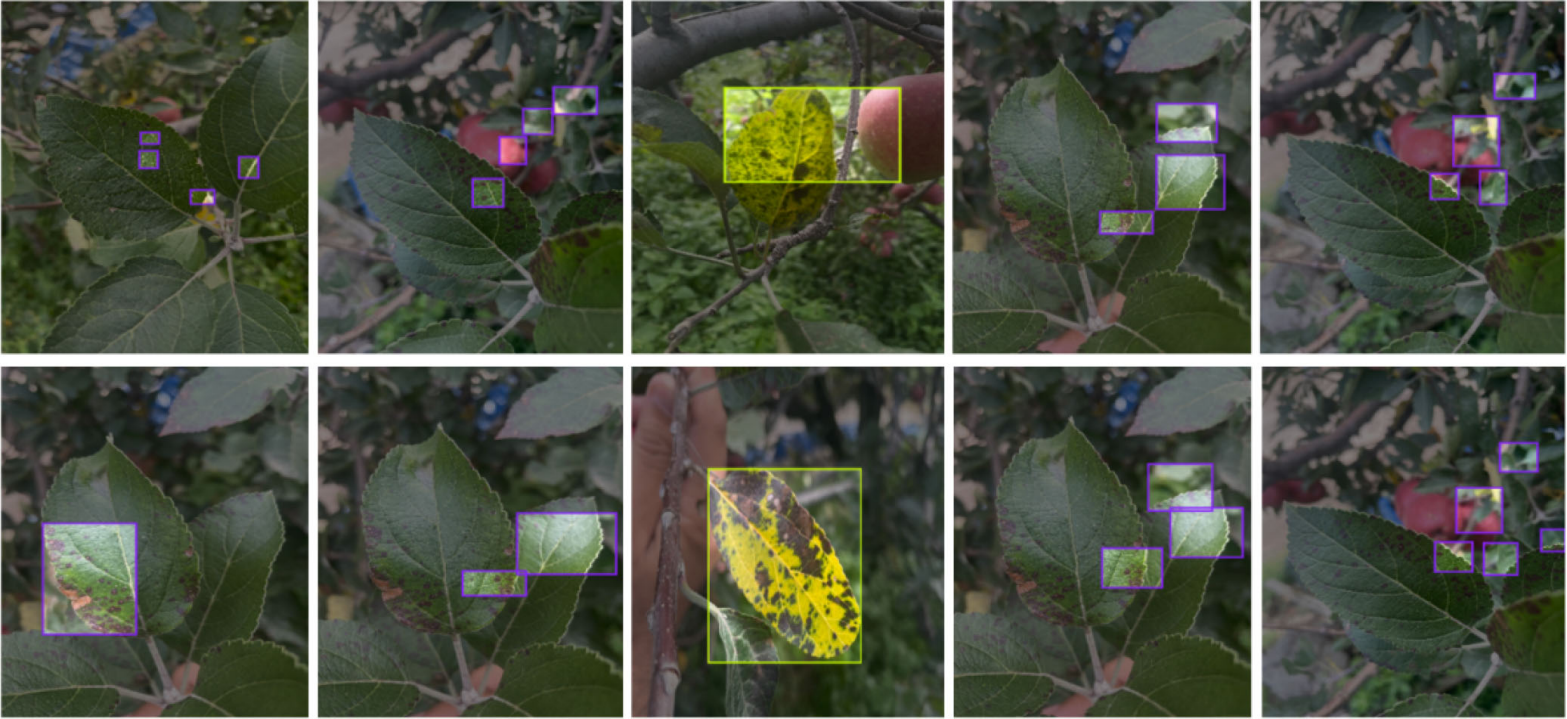
Performance of the YOLO-Leaf model on powdery mildew.

We validated our model on a field dataset that contains a significant amount of powdery mildew. **Figure 12** shows the detection results of our model, demonstrating its effectiveness and generalization capability in real-world conditions.

### 4.7 Ablation study

As shown in **Table 6**, the results of the ablation experiments demonstrate the contribution of each component to the overall performance of our method. By analyzing the table, it is clear that the inclusion of DSCon, BiFormer, and IF-CIoU individually and in combination significantly impacts the mAP50 and mAP50-95 metrics. For instance, the baseline model YOLOv8n, which does not include any of these components, achieves an mAP50 of 83.26% and mAP50-95 of 85.37%. When DSCon is added (YOLOv8n-a), the mAP50 slightly decreases to 82.32%, and mAP50-95 drops to 84.33%, suggesting that DSCon alone does not significantly improve performance. On the other hand, adding BiFormer (YOLOv8n-b) results in an mAP50 of 81.26% and mAP50-95 of 85.67%, showing a slight improvement in mAP50-95 but a decrease in mAP50. Adding IF-CIoU (YOLOv8n-c) increases the mAP50 to 85.34% but reduces mAP50-95 to 82.15%. When DSCon and BiFormer are combined (YOLOv8n-d), the performance significantly improves, with mAP50 reaching 90.75% and mAP50-95 at 87.66%. Similarly, the combination of BiFormer and IF-CIoU (YOLOv8n-e) further enhances the results to an mAP50 of 91.33% and mAP50-95 of 90.15%.

**Table 6:** Experiment results for each component.

Method	DSCon	BiFormer	IF-CIoU	mAP50	mAP50-95
YOLOv8n	✗	✗	✗	83.26	85.37
YOLOv8n-a	✓	✗	✗	82.32	84.33
YOLOv8n-b	✗	✓	✗	81.26	85.67
YOLOv8n-c	✗	✗	✓	85.34	82.15
YOLOv8n-d	✓	✓	✗	90.75	87.66
YOLOv8n-e	✗	✓	✓	91.33	90.15
Ours	✓	✓	✓	93.88	95.69

Our method, which integrates all three components (DSCon, BiFormer, and IF-CIoU), achieves the highest performance with an mAP50 of 93.81% and mAP50-95 of 95.69%. This comprehensive combination demonstrates the significant advantage of using all components together.

## 5 Conclusion

In this paper, we propose a novel apple leaf disease detection model, YOLO-Leaf, which integrates three key technologies: DSConv, BiFormer, and IF-CIoU. These technologies work in close synergy to effectively address the detection challenges posed by different apple leaf diseases. Specifically, the combination of DSConv, BiFormer, and IF-CIoU allows the model to better identify the dispersed lesions of rust disease on leaf surfaces and accurately capture the changes along the edges and veins in scab disease. IF-CIoU further optimizes the alignment between bounding boxes and actual lesion areas, particularly excelling in handling the variable shapes and sizes of spots in leaf spot disease. Experimental results demonstrate that YOLO-Leaf outperforms existing models like YOLOv3, YOLOv4, and YOLOv8 across multiple evaluation metrics, fully validating the effectiveness and advancement of our approach.

Despite YOLO-Leaf’s excellent performance across various metrics, the model still has some limitations. First, when processing images with complex backgrounds, the detection accuracy of YOLO-Leaf decreases. This is mainly because complex backgrounds may interfere with the identification of disease regions, leading to false positives or missed detections. Second, the performance of YOLO-Leaf in detecting small disease spots still needs improvement. Although we introduced dynamic snake convolution and a dual-layer routing attention mechanism to enhance feature extraction capabilities, recognizing extremely small disease spots remains challenging, which limits the model’s application scope to some extent. In the future, we plan to improve the model in the following aspects. First, we will further optimize YOLO-Leaf’s feature extraction module, particularly focusing on detecting complex backgrounds and small disease spots, exploring more effective feature extraction methods. Second, we will expand the dataset’s size and diversity, including introducing more field-captured images and different types of disease images, to enhance the model’s generalization ability. Additionally, we will explore multimodal data fusion techniques, combining image data with other types of data (such as temperature, humidity, etc.) to improve the accuracy and robustness of disease detection. This study not only provides an efficient solution for apple leaf disease detection but also offers new ideas and methods for research and applications in the field of agricultural disease detection.

## Data Availability

The original contributions presented in the study are included in the article/supplementary material. Further inquiries can be directed to the corresponding author.
